# The Potential of Waste-Derived Sorbents for Absorbing Petroleum Substances in Firefighting Operations

**DOI:** 10.3390/ma18163752

**Published:** 2025-08-11

**Authors:** Justyna Gniazdowska, Anna Rabajczyk, Tomasz Wilczyński, Daniel Małozięć

**Affiliations:** 1 Laboratory of Fire Extinguishing Agents and Equipment–BU, Scientific and Research Centre for Fire Protection-National Research Institute, Nadwiślańska 213, 05-420 Józefów, Poland; jgniazdowska@cnbop.pl (J.G.); twilczynski@cnbop.pl (T.W.); 2 Study Works and Research Projects Department, Scientific and Research Centre for Fire Protection-National Research Institute, Nadwiślańska 213, 05-420 Józefów, Poland; 3 Deputy Director for Research and Development at Scientific and Research Centre for Fire Protection-National Research Institute, Scientific and Research Centre for Fire Protection-National Research Institute, Nadwiślańska 213, 05-420 Józefów, Poland; dmaloziec@cnbop.pl

**Keywords:** sorbents from waste, oil spills, sorption capacity

## Abstract

The development of industry and technology, despite making everyday life easier, generates large amounts of various wastes that negatively affect the environment. Unexpected leaks of substances such as oils, petroleum substances, and chemicals also contribute to the degradation of aquatic and terrestrial ecosystems. Long-term effects of environmental pollution require the development of advanced materials and technologies to collect and neutralize pollutants. Sorbents obtained from waste, including banana peels, coconut fibers, and polyurethane foams from recycling the thermal housing of refrigeration devices, allow a reduction in the amount of generated waste and the development of appropriate sorbents. This work focuses on comparing the sorption and neutralization properties of these materials for two types of oil, machine and diesel, and the possibility of using them in rescue and firefighting operations conducted by firefighters. The results obtained indicate that the viscose–cellulose sorbent and the polyurethane foam sorbent are characterized by better performance parameters than sorbents from coffee grounds or coconut fibers. The best parameters were obtained after the first 10 min of the sorbent–contaminant reaction, whereas in the case of contamination with machine oil, the absorption capacity was better than for diesel oil for each sorbent subjected to analysis.

## 1. Introduction

The development of technology in various areas, including the automotive, textile and electronics industries, contributes to an increased quality of life for humans. Unfortunately, in addition to improving quality of life, it also leads to the deterioration of the quality of the environment, which is a serious problem for our planet [1]. As a result of anthropogenic activity, various substances are introduced into the environment, sometimes with very different physicochemical characteristics, such as heavy metals, alkali metals, oils, and plastics. Some of the most important sources of oil- and petroleum-derivative pollution include industrial factories, combustion engines, refineries, or spills during the extraction and transport of crude oil or road accidents [2,3]. In connection with the above, the interest in searching for modern solutions for neutralizing pollutants is constantly growing. In the case of liquid pollutants, insoluble in water, such as petroleum derivatives, methods and materials are necessary to collect oily substances from solid surfaces and/or watercourses and then dispose of them [4,5]. The preferred techniques for removing pollutants are sorption processes. It is an easy and cheap method that is environmentally friendly and can work in the place where the pollution occurs, without the need to use additional solutions.

Sorbents are porous materials or a mixture of these materials that have sorption capabilities, and these processes may also be accompanied by various chemical reactions, which often support the process of neutralizing contamination [4,6]. They are mainly used in places such as industrial plants, warehouses, and petrol stations. They are also part of the equipment of the State Fire Service, among others, for removing contamination after road collisions.

**Figure 1:**
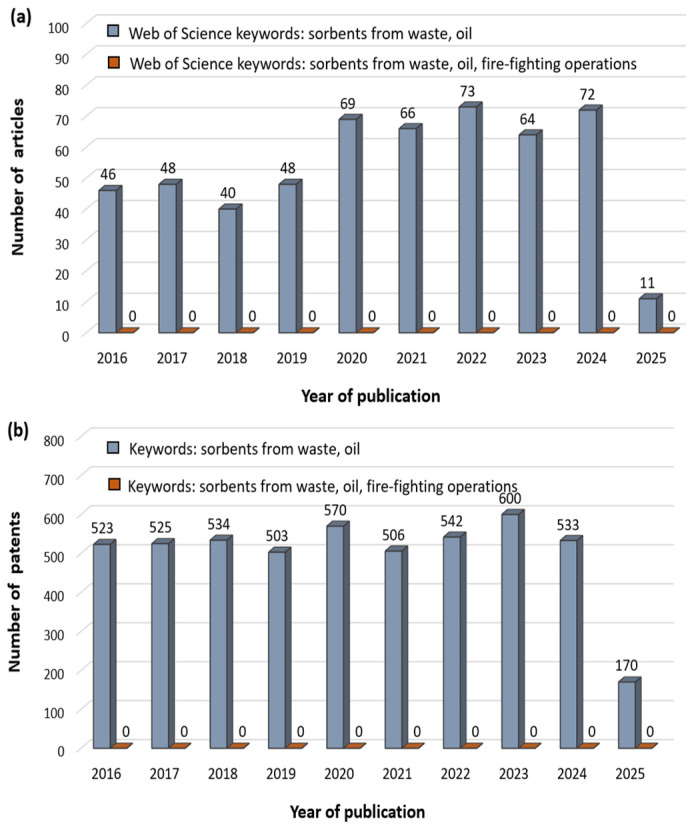
Number of articles (**a**) and patents (**b**) published in the years from 2016 to March 2025 analyzed for the following keywords: recycled sorbents/sorbents from waste, and oil, with the scope of the search as follows: Subject-title, abstract, author keywords, and Keywords Plus. Source: Web of Science database and patent databases such as Espacenet, GooglePatents, Patentscope, and European Patent Register (access: 8 April 2025).

An increasingly large group of sorbents are those produced by recycling materials, usually considered production waste. This paper will present the most important information on the conditions of use of selected materials based on a review of the literature and available patent databases such as Espacenet, Google Patents, Patentscope, and European Patent Register (Figure 1). Over the last few years, i.e., from January 2016 to March 2025, the time period covered by the analysis, dynamic development can be observed in the field of the use of sorbents obtained through recycling in the neutralization processes of oily substances, including petroleum derivatives. This is evidenced by the continuous increase in scientific publications in this area (Figure 1a).

However, taking into account the number of technical solutions protected by patent law (Figure 1b), a much greater activity is observed in the production of sorbents from waste and their use in the removal of oil contamination. In turn, no data has been reported regarding the management of contamination generated during firefighting operations, for example, to remove oil spills, based on sorbents obtained through recycling. 

## 2. General Characteristics of Sorbents

Sorbents are divided according to their origin into natural sorbents, including organic and inorganic sorbents, and synthetic sorbents. Natural organic sorbents are easily available and persist well in water but are usually disposable. After use, this material can be disposed of by incineration. Despite this disadvantage, they are considered the most effective [4].

Other criteria, including those included in various norms and standards around the world, include the division of sorbents according to their applicability in a given environment, hydrophobic properties, or sorbent structure (Table 1). 

Contamination with petroleum-derived and oily substances significantly impacts environmental quality. Considering the location of contamination and the properties of the oily substances, access to tools that allow for their removal from the environment is essential. In combating this type of contamination, materials derived from waste are gaining increasing importance in the sorbent market, including polyurethane foam, coffee grounds, coconut fiber, banana peels, sawdust, and waste from the production of tissues (cellulose waste, cellulose–viscose waste) or sugarcane bagasse. One approach involves modifying existing materials using oil waste, while another involves using solid waste through selected physical processes (e.g., drying, roasting, grinding) and/or modifying it with other substances. An example of the former approach is the modification of coconut-polyurethane (PU) foam (CCF) materials through a conventional foaming process using varying amounts of coconut oil-derived polyol (CODP) in the PU matrix [12]. The latter approach involves sorbents obtained from various plant fibers, mixed leaf debris, mixed sawdust, sisal (Agave sisalana), coconut fiber (Cocos nucifera), sponge gourd (Luffa cylindrica), and silk thread [13].

Taking into account various criteria for dividing sorbents, the most important elements of assessing a given material as a sorbent seem to be its utility parameters. The sorption capacity of a sorbent, which is the basic measure of its usefulness, is the mass of the absorbed substance (sorbate) to the mass of the loose sorbent that absorbed it. It is assumed that the higher the sorption value of the sorbent, the lower the amount that should be used, which is directly related to reducing the costs of purchasing such a material, as well as its disposal [14]. The aim of this study was to determine the potential of sorbents obtained through recycling in the work of fire departments and units involved in the protection and elimination of petroleum-derived contamination. The focus was on comparing the physicochemical properties of sorbents obtained through recycling, which are important for fire department operations and determine the effectiveness of capturing various oil contaminants that may arise, for example, during a breakdown or a car accident. The contact time of the sorbent with oil was verified using various waste-derived materials and various oils to verify the sorption capacity of these materials against various contaminants. The data obtained during this work can provide a basis for developing more adequate guidelines for assessing sorbents used in firefighting operations.

## 3. Materials and Methods

### 3.1. Reagents and Materials 

**Figure 2:**
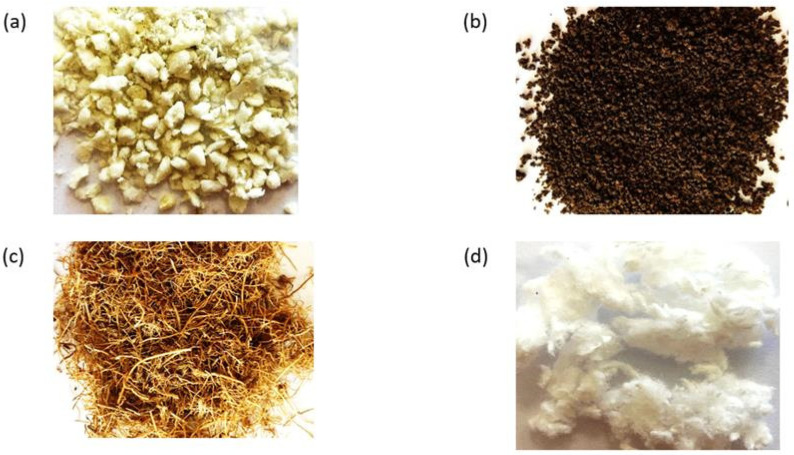
Materials used as sorbents: (**a**) polyurethane foam, (**b**) coffee grounds, (**c**) coconut fiber, (**d**) viscose–cellulose sorbent.

The following waste materials were used for the tests:

1. Polyurethane foam (Figure 2a): hard, degassed polyurethane foam (PU) from recycling the thermal casing of refrigeration devices, with varying degrees of flammability; it was ground to obtain materials with varying degrees of fragmentation;
2. Coffee grounds (Figure 2b): a mixture of Arabica and Robusta, MKCafe Select, obtained from a pressure espresso machine with grinding using a ceramic grinder; wet coffee ground disks were dried in a temperature chamber for 24 ± 0.5 h at 55 ± 0.5 °C and then poured onto a flat tray of 26 × 20 cm for another 24 ± 0.5 h at 20 ± 5 °C and dried in the open air;
3. Coconut fiber (Figure 2c): ground coconut fiber mixed with ground coconut shell fragments;
4. Viscose–cellulose sorbent (Figure 2d): viscose–cellulose nonwoven fabric with a composition of 50.3% viscose and 49.7% cotton, waste from the production of wet wipes and a material in fibrous form.

The tests used operating fluids that are most commonly used to drive compression-ignition vehicles. They are also used to lubricate lightly or medium-loaded working elements of industrial machines and devices, piston and slide bearings, and open mechanical transmissions exposed to low temperatures. The following were used for the research:

- Diesel oil compliant with EN 590 (ORLEN OIL Sp. z o.o., Gdańsk, Poland): oil intended for compression–ignition engines; density at 15 °C of 838.0 kg/m^3^, flash point of 65.0 °C, cetane index of 5.1, viscosity at 40 °C of 2,686 mm^2^/s, sulfur content of 6.1 mg/kg, and cetane number of 53.5;
- Jasol Machine Oil AN Z (46Z) machine oil (Flukar Ltd., Katowice, Poland): oil intended for lubrication of lightly or medium-loaded working elements of industrial machines and devices, piston and slide bearings, and open mechanical transmissions exposed to low temperatures; it is a mixture of mineral base oils and an additive improving low-temperature properties—heavy paraffinic distillates, hydrotreated (petroleum)—unspecified base oil, content 100% by weight; pour point < −24 °C; flash point > 200 °C; density at 15 °C of approx. 0.88 g/cm^3^; and kinematic viscosity at 40 °C of 28.8–74.8 mm^2^/s [15].

### 3.2. Research Equipment

We used conical sieves made of 0H18N9 mesh with the following dimensions: sieve 1: base diameter 70 mm, height 75 mm, mesh with mesh size 0.25 mm with lids; sieve 2: base diameter 100 mm, height 105 mm, mesh with mesh size 0.25 mm with lids (the Laboratory of Fire Extinguishing Devices and Agents of the BU, Scientific and Research Centre for Fire Protection-National Research Institute, Poland). We used the Radwag scale (Radwag, Radom, Poland), indication range 0.5–2100 g, class e = 0.1 g. The Hosokawa Alipine Aktiengesellschaft 200 LS-N (Augsburg, Germany) air sieve was employed. We used the KWT Włocławek thermometer (KUJAWSKA WYTWÓRNIA TERMOMETRÓW, Włocławek, Poland), with a range from −1.2 to 51.1 °C, with an accuracy of 0.2 °C, and the OLYMPUS BX51 system microscope with a camera (OLYMPUS OPTICAL CO. (EUROPA) GMBH, Hamburg, Germany).

### 3.3. Methodology and Calculations

The analysis of selected materials as sorption materials that can meet the requirements of the fire service included 5 performance parameters, including oil absorption capacity, sieve analysis, chemical inertness, buoyancy, and bulk density (Table 2).

**Figure 3:**
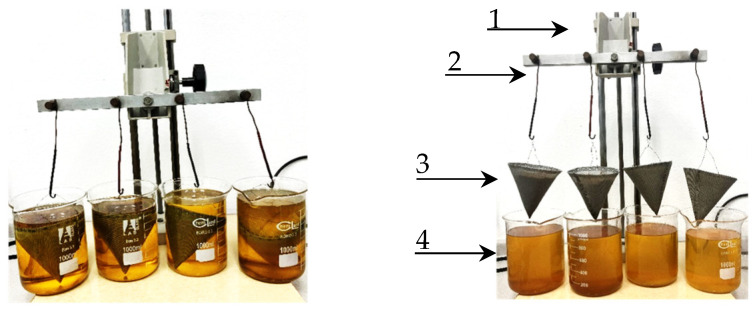
Oil absorption capacity testing station (system components: 1. Stand; 2. Holders; 3. Sieves with mesh sizes specified in the procedure; and 4. 1000 mL beakers).

The oil absorption capacity test was conducted using the method developed by the Laboratory of Fire Extinguishing Devices and Agents of the BU, Scientific and Research Centre for Fire Protection-National Research Institute (in short: BU), a method accredited by the PCA (Polish Centre for Accreditation) with the AB060 [16], based on the Westinghouse method. The work in this area was carried out using a dedicated stand for absorption capacity testing prepared by the Laboratory of the BU (Figure 3).

The influence of time on the assessment of the effectiveness of the absorption capacity of both diesel and machine oil was verified by modifying the developed Westinghouse method additionally by adding soaking and draining times of 10, 30, and 60 min, respectively. Based on the mass measurements before and after sorption of the operating fluid, the amount of absorbed medium (expressed in %) was calculated.

The analysis of individual parameters was performed four times for each material using the appropriate oil under study. 

## 4. Results and Discussion

Among the basic tools necessary for the implementation of fire brigade tasks in the field of collecting contaminants are sorbents. However, their use is conditional and depends on many factors, including the type and size of the surface on which the contamination occurs, atmospheric conditions, the type of contamination, and the type of sorbent.

In the operations of fire departments and other units responsible for neutralizing contamination, operational parameters are crucial, determining the speed and effectiveness of actions to protect the environment and human property. Sorbents, in addition to their utility features, should also be easy to use. One method is to manually spread a given sorbent; another is to spread it mechanically, using seeders or brushes, for example. However, many sorbents are loose bulk materials and fine-grained, which makes them difficult to use in windy conditions. When carried by the wind, they can contribute to health risks in people involved in the incident if they inhale sorbent particles. Therefore, it is important that sorbent materials are environmentally friendly and do not pose a threat to the health and life of people involved in the incident. Fiber compressed into a mat makes the material easier to handle and reduces the possibility of dust formation. The use of binding materials can improve the durability and ease of handling of the product, but non-biodegradable binding materials reduce its ecological value [17].

Technical and operational requirements that sorbents used by fire protection units must meet include, among others, requirements regarding absorbency, buoyancy (for sorbents used on water), reactivity, grain size, and bulk density. Some studies also indicate the need to take into account the degree of roadway roughness restoration by the sorbent used [18]. The results of the static friction coefficient tests conducted by Sobolewski et al. [18] indicated that the use of sand to collect fuel from the roadway surface did not restore its original roughness, because the surface was still contaminated. It was also found that leaving the scattered sorbent on the roadway surface did not provide satisfactory results in terms of improving the surface adhesion. The restoration of the original friction coefficient and thorough cleaning of the surface were only achieved after collecting the sorbent with the absorbed fuel, with better capabilities observed for the fine-granulated sorbent [18].

**Figure 4:**
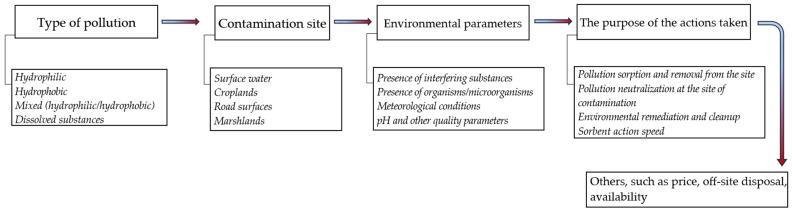
Example of sorbent selection path depending on the contamination.

The criteria for assessing the suitability of a given material as a sorbent for pollutant neutralization take into account various elements (Figure 4), including the following:

- The material’s absorption capacity for the selected pollutant (hydrophobic substances are better suited for absorbing oily pollutants);
- Bulk density, which determines its suitability for use in open environments (materials that are too light can be carried long distances by the wind);
- Costs resulting from the multiple processing stages, a complex production line, and modification/biomodification determine the price of the sorbent (the more complex the technological process, the higher the cost of producing the material);
- Post-use management, which influences further processes and increases the cost of using the material.

Taking the above into account, before selecting a sorbent, it is essential to analyze the advantages and disadvantages of a given material in terms of its potential practical application.

### 4.1. Physicochemical Parameters of Sorbents Obtained from Waste

The test methods confirming the fulfillment of the utility requirements are not described by any Polish standard. The Laboratory of Fire Extinguishing Devices and Agents (BU) CNBOP-PIB has developed its own procedures for verifying whether a given sorbent meets the requirements of the relevant legal acts [13], which were used in this study. For each of the sorbents, tests were carried out for five parameters, grain composition, chemical passivity, buoyancy, and bulk density, in accordance with the methodologies specified in point 3.3, and the results obtained are presented in Table 3.

Sorbents must be chemically passive, because the chemical reaction between these media during the neutralization of oil contamination may pose a threat to both the people involved in the neutralization and the environment. The reaction may be strongly exothermic, which may even lead to the risk of explosion, or it may lead to the formation of new compounds with toxic properties. The first step is to verify whether the selected materials were chemically passive. The results of the chemical inertness tests showed that all of the tested samples of sorption materials were chemically passive. The sorbents were not found to react with the operating fluids used in the tests, and they did not change their color or form. There was also no change in the temperature of the absorbed medium or its color change during the tests. This parameter defines the nature of sorption processes occurring in the oil–sorbent system. The process observed, regardless of the system analyzed, is based on physical sorption, involving weak van der Waals interactions occurring between the adsorbate molecules and the solid surface.

**Figure 5:**
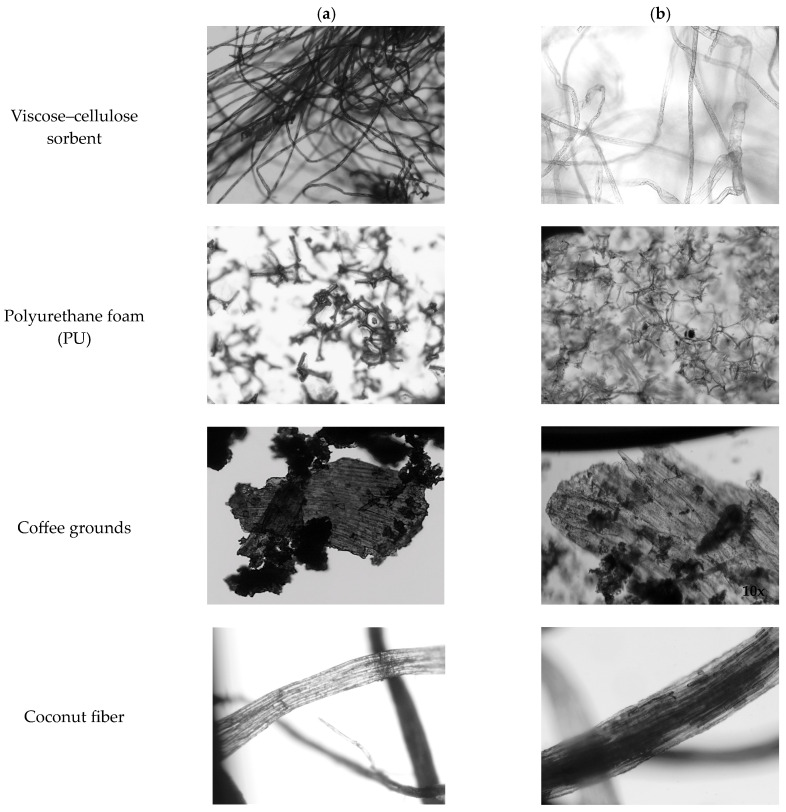
Sorption materials: pure (**a**) and after the machine oil absorption process (**b**) (2048 × 1532, Olympus Soft Imaging Solutions SC30, 1/500 s).

Microscopic observation (Figure 5) allowed us to determine the structure of individual materials and to observe the oil sorption process. The viscose–cellulose sorbent and coconut fiber are characterized by long fibers, while the open-cell polyurethane foam is characterized by irregular, polyhedral, closed or torn, porous structures. Coffee grounds have a different structure, in the mass of which there are both irregular, compact grains and large surfaces.

Waste material is heterogeneous, originating from various points in the technological process, use, or storage. This makes these materials inhomogeneous and difficult to reuse. Grain size and microscopic analysis also reveal a lack of homogeneity in a given sorbent. Cutting, crushing, grinding, and drying processes allow us to obtain a finer structure. However, because the starting material itself is not homogeneous and is often even defective, obtaining a sorbent that is uniform throughout its entire volume is impossible. It should also be noted that in the context of practical applications for neutralizing contaminants during rescue operations, there are no guidelines for determining the material’s structure or porosity. Important parameters include the following:

- Chemical inertness, which allows for sorption to be based on physical processes;
- Buoyancy, which allows us to determine whether the material is suitable for use on water or paved surfaces;
- Bulk density, which allows us to determine whether the material is lightweight and will be carried by the wind during use or whether it is heavier and poses no such risk.

The differentiation of the structure affects the oil sorption mechanism observed for individual sorption materials. 

**Figure 6:**
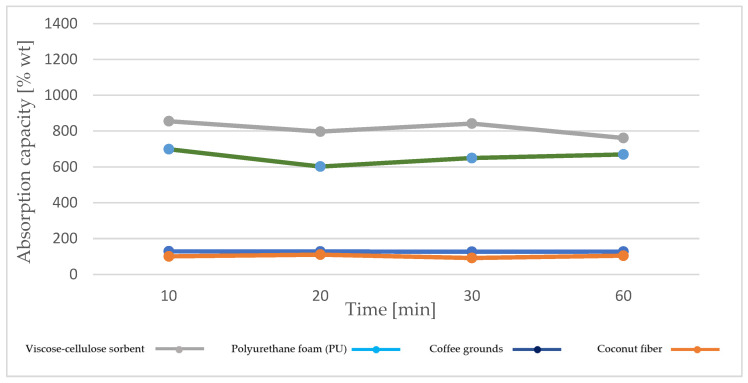
Diesel oil absorption capacity of recovered sorbents, taking into account different soaking and draining times.

**Figure 7:**
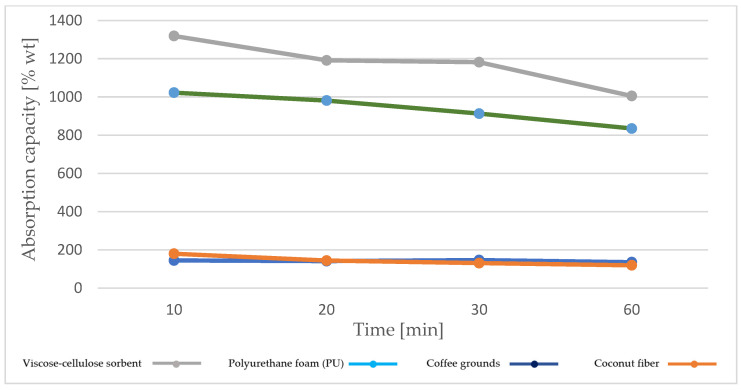
Absorption capacity of AN Z (46Z) machine oil by recovered sorbents, taking into account different soaking and draining times.

In the case of the viscose–cellulose sorbent, where there are cellulose and viscose spaces created as a result of cellulose processing, the oil is sorbed both on the surface of the material and penetrates the interior of the structure. Two sorption paths mean that in the case of this material, the oil absorption capacity is the best (Figure 6 and Figure 7). In the case of polyurethane foam, on the other hand, the oil drops are bound by the irregular walls of the crushed material, and in this way, the oil is retained by the sorbent. Coffee grounds, which consist of both irregular coffee particles and particles with an expanded sorption surface, retained the oil by sorbing the contaminant on the surface. Narrow and hollow fibers, with thick walls made of cellulose, which characterize coconut fibers, allowed us to observe the slow, gradual penetration of oil into the fiber interior while pushing out the air present in the fiber spaces. Probably for this reason, the oil absorption capacity was the lowest in the case of this sorption material. Abdelwahab et al. [19] demonstrated that the adsorption process can be described as a monolayer coating, which would allow the use of the Langmuir adsorption isotherm to characterize the process. However, for complex mixtures such as diesel fuel or crude oil, the use of the Langmuir or Freundlich isotherms is difficult. This is due to the fact that not all components of petroleum substances are subject to these isotherms as single solutes [20].

### 4.2. Oil Absorption Capacity

#### 4.2.1. Diesel Fuel Absorption Capacity

Road accidents, as well as work in car workshops, are often accompanied by leakage of operating oils. It is necessary not only to prevent their further spread but also to effectively remove the hazard that may contribute (or does contribute) to environmental pollution. The most common operating fluids emitted as a result of road accidents are diesel oil, petrol, machine oil, and coolants [14,21,22]. Diesel oil for compression–ignition engines is used as a reference substance in sorbent tests, in accordance with EN 590. Based on the conducted tests, it was found that the highest capacity for absorbing diesel oil is achieved by materials such as viscose–cellulose sorbent and polyurethane foam (Figure 6). This is related to the structure of the material. Both materials have large pores in their structure, which allow for the penetration of a larger amount of the absorbed medium. It should be noted, however, that the largest amounts were absorbed within the first 10 min, and with the extension of the oil soaking time and the corresponding extension of the dripping time, a tendency can be observed for the value of this parameter to reduce.

A different situation was observed in the case of natural sorption materials, such as coffee grounds or coconut fiber with an admixture of nut shells. In both cases, although the capacity is several times lower compared to sorbents based on polymer structures such as cellulose or polyurethane, the absorption capacity remains at a comparable level regardless of the soaking time and ranges from 128.74 to 126.63% for coffee grounds and from 91.92 to 110.5% for coconut fiber.

#### 4.2.2. Machine Oil Absorption Capacity AN Z (46Z)

Machine oil is an equally important contaminant, especially in the case of incidents in workshops, factories, and logistics centers. It is a substance released from various systems and mechanisms. Therefore, Jasol Machine Oil AN Z (46Z) was selected for the study, intended for lubrication of lightly or medium-loaded working elements of industrial machines and devices, piston and slide bearings, and open mechanical gears exposed to low temperatures. This oil is characterized by different parameters, in terms of density, viscosity, and flash point, from diesel oil. Based on the studies conducted, it was found that, similarly to diesel oil, the highest absorption capacity of machine oil AN Z (46Z) is achieved by polymer-based materials, such as viscose–cellulose sorbent and polyurethane foam (Figure 7). However, also in the case of machine oil, with the extension of the oil soaking time and the corresponding extension of the dripping time, there is a gradual decrease in the value of this parameter. In the case of machine oil, the downward trend is more intense.

In the case of coffee grounds and coconut fiber, similarly to diesel oil, the absorption capacity is several times lower than for polymers and remains at a comparable level. Comparing the sorption capacity of the analyzed materials in relation to machine oil and diesel oil, it should be noted that in the case of machine oil, higher absorption capacity values were obtained for each sorbent. 

#### 4.2.3. Possibility of Using Waste-Based Sorbents

In recent years, many researchers have become interested in the use of agricultural waste or by-products in the process of adsorption of pollutants, including primarily petroleum substances. These materials have many advantages and are an alternative to synthetic sorbents, mainly due to biodegradability [3]. However, in order to minimize post-production waste, other waste is also used, such as polyurethane foam or coconut fiber, which may originate, for example, from the production of sleeping mattresses (Table 4).

Porosity, structure, and the presence of active sites are among the parameters that allow for the determination of the potential properties of a material as a sorbent. However, it should be noted that in the case of oil sorption, according to the literature data, fiber porosity and oil sorption capacity are correlated, but only to a certain extent [27]. Rengasamy et al. [28] demonstrated that for fibers with porosity greater than 0.98 (non-fiber volume fraction), capillary forces are overcome by gravity, which significantly reduces oil sorption capacity. It has also been shown that a rougher surface typically has a greater capacity for retaining absorbed oil. A rough fiber surface, resulting, for example, from the presence of fine hairs on the surface, increases the surface area for oil adhesion [29]. Considering the need to combine practical properties with waste management, the most important parameters are practical ones, which directly indicate whether a given material can be used in situations contaminated with oily substances and whether it will be easy to use and environmentally friendly. The main consideration in such a situation is the possibility of leaving the sorbent in its place of use.

Sorption materials made from waste often have better performance parameters than synthetic sorbents specially produced and dedicated to a given pollutant. For example, Suni et al. [17] studied the absorption capacity of a natural, slowly biodegradable, hydrophobic sorbent such as cotton grass fiber, and compared its ability to absorb oil and water with that of a synthetic, commercial sorbent. The results of the studies conducted showed that cotton grass fibers are characterized by better performance parameters in terms of both absorption capacity and absorption rate [17]. 

Analysis of available materials obtained from waste that can act as sorbents for oil contaminants indicates that the best utility properties are possessed by cotton, viscose–cellulose sorbent, and barley straw, the structure of which is based on high cellulose content. Wastes such as coconut fiber or peels contain cellulose, which can affect their efficiency in removing oil contaminants. It should be noted, however, that the structure of the material is not the only important aspect in assessing the value and significance of a given sorbent [30,31,32]. The work conducted by M. Hussein et al. [25] showed that the oil sorption capacity depended on the sorption time and oil temperature or the thickness of the film formed on the seawater surface. For this purpose, barley straw fibers produced in Egypt were used, and the sorption process was carried out in a simulated sea bath containing crude oil. In turn, the results of the research conducted by El-Din et al. [3], in which skins from the study were used, indicate that the surface properties, type of oil, oil film thickness, sorption time, temperature, and salinity are important for the oil sorption process. The best sorption effects of weathered crude oil were obtained with an average particle size of 0.3625 mm, temperature of 25 °C, sorption time of 15 min, concentration of 3.5% of artificial sea water, and oil film thickness of 5 mm. The results of the research by Abdelwahab et al. [19] showed that the adsorption capacity of diesel oil, crude oil, and vegetable palm fibers increases with time, oil film thickness, temperature, and particle size, while it decreases with the mass of the adsorbent [19]. 

According to statistical data obtained from the Main Headquarters of the State Fire Service in Poland, approximately 828,144.3 kg of sorbents were used in the years 2015-2022 [33]. In rescue and firefighting operations, firefighters use commercially purchased sorbent resources with valid approval certificates issued by appropriate certification bodies, such as CNBOP-PIB. In situations where standard sorbents are not available or have been exhausted, it is possible to use dried and crushed waste from plant production, such as bran, cereal chaff, ground tree bark, leaves, needles, reed, ground corn cobs, nutshells, sea grass, or waste paper. The results of the tests indicate that the materials analyzed meet the relevant requirements and can be used by fire departments to eliminate selected contaminants in various systems (Table 5).

### 4.3. Disposal of Sorbents

The collection of oil and oil-derived contaminants generates a problem with their subsequent disposal. Used sorbents are treated as waste from the oil and gas industry [34,35] and are disposed of as hazardous waste. The process of their disposal may consist of, among other stages, burning the contaminants at high temperature and increased oxygen content in order to oxidize all contaminants to dioxide and water [36]. During the burning process, a significant amount of volatile compounds, which belong to the group of harmful substances, are released into the atmosphere [14]. Some sorbents subjected to such treatment can be reused in accordance with their intended purpose or used as building materials. However, the described process of regeneration of used sorbents is expensive, and for this reason, other methods are sought that allow for safe disposal and, best of all, reuse of sorbents using biological methods that do not require high temperatures. 

Hussein et al. [25] used a mechanical press to remove the absorbed diesel oil from the applied sorbent, which is barley straw fiber. Such procedures with the used sorbent make it possible to reuse the sorbent, especially in the case of limited water absorption during contamination neutralization, resulting from the coating of barley straw fibers with wax. The results suggest that it is possible to replace commercial synthetic oil sorbents in cleaning oil spills with agricultural residues, which may be beneficial when taking into account other advantages, such as biodegradability [25]. Also, Alaa El-Din et al. [3] used mechanical oil removal first and then n-hexane extraction, which maintained the stability of the fiber and chemical composition of banana peels. 

Another method of disposal is the biodegradation of the used sorbents through biological decomposition of petroleum derivatives and other organic pollutants in a humid environment with access to air. However, this process is long-term, requiring many processes, including mechanical ones, such as mixing. It should be added that if the concentration of the hazardous substance is lower than indicated in the standard, the material can be used for final storage [23]. Such a procedure requires constant analysis and monitoring of the process, but it allows for the minimization of hazardous waste and the limiting of the combustion process, which produces, among other emissions, CO_2_. 

The sorption materials covered by the research, with the exception of polyurethane foam, are based on natural materials, which means that they can be biodegraded. For example, viscose–cellulose material and coconut fiber are materials used to strengthen soil layers. They are used as materials on the surface of which microorganisms can settle, meaning that the process of biological decomposition of the material and adsorbed pollutants can take place. In the case of natural sorbents, such as coffee grounds, coconut fiber, and viscose–cellulose material, management also includes biodegradation or use in remediation processes. However, the form and composition of a given waste influences the time and process of biodegradation itself, including the type of organisms determining biological decomposition. Hubbe et al. [37] have shown that the form of cellulose, such as cotton, paper pulp fibers, or microcrystalline cellulose, determines the time, degree, and complexity of the cellulose biodegradation process. Viscose fibers, which are wood-based cellulose fibers, are also fully biodegradable. However, it has been shown that the limited degradation of treated and modified cellulose may be due to the hydrophobic surface, which discourages strong biofilm development [38]. The use of coconut fiber-based waste was studied, among others, by Jishnu et al. [39]. The results of the analyses conducted indicate the possibility of using coconut fibers for soil reinforcement. Natomas Mir and Bawa [40] found that the introduction of coconut fiber into clay soil increases the friction forces between soil particles and reinforcing fibers, so this waste can be used in embankments and soil consolidation for roads.

Thanks to these materials, the number of sorbents requiring special treatment in the disposal process can be reduced. This is important, because very often contamination covers a large area, and collecting the used sorbent and then burning it can be difficult and expensive. One direction for sorbent development should be the consideration of their disposal methods after use, including neutral/environmentally friendly processes.

## 5. Conclusions

Growing pro-ecological awareness and increasingly restrictive requirements in the field of environmental protection mean that in many areas, actions are being taken to recover various materials that can act as sorbents for selected pollutants. Sorbents obtained from waste, including banana peels, coconut fibers, and polyurethane foams from recycling the thermal housing of refrigeration devices, allow us to reduce the amount of waste generated while maintaining the effectiveness of pollutant neutralization. 

Some of the main criteria for assessing the efficiency of a material in the pollutant neutralization process are as follows:

- The ease and speed of preparing sorbents (simple processes such as crushing, grinding, and drying)—each additional process generates costs and the potential for further waste generation;
- The possibility of easy and quick use, without negative impacts on humans and the environment;
- Methods for disposing of used sorbents that are simple, energy-efficient, and environmentally safe, preferably based on biodegradation.

All tested sorbents obtained from waste meet the requirements in terms of functional characteristics and can therefore be used by fire brigades to collect oil and petroleum pollutants. The results of our research indicate that the use of different oils for testing gives different results for a given sorption material obtained from waste, whereby the following is true:

- The best operating parameters are obtained for the viscose–cellulose sorbent for both oils, with higher absorption capacity values obtained for machine oil;
- The absorption capacity values for diesel oil are lower than for machine oil in the case of all sorption materials analyzed;
- The viscose–cellulose sorbent and polyurethane foam are characterized by higher absorption capacity for both oils compared to sorbents based on coffee grounds and coconut fiber;
- Sorbents based on coffee grounds and coconut fiber show stability over time in terms of absorption capacity for both types of oil.

It should be noted that for all systems, the first 10 min of sorption were the most important. After this time, the absorption capacity decreased, as in the case of the viscose–cellulose sorbent and polyurethane foam, or remained at a comparable level, as in the case of sorbents based on coffee grounds and coconut fiber. Conducting research on the contaminant-specific sorbent system obtained from waste should provide a basis for further work on minimizing the negative environmental impact and optimizing the effectiveness of materials obtained this way. Depending on the type of sorbent and the type of contaminant, the effectiveness of contaminant neutralization may be different. However, for the actions undertaken by fire departments and other units responsible for neutralizing oil contamination, it is necessary to develop guidelines with a broader scope than previously available. Introducing a wider range of analyses to the scope of requirements for sorbents, taking into account different types of pollutants, would allow for a better selection of materials for such applications.

## Figures and Tables

**Table 1:** Division of sorbents, taking into account the requirements of selected standards introduced around the world.

Standard	Division of Sorbents	Ref.
ASTM F726-12: Standard Test Method for Sorbent Performance of Adsorbents, Annual Book of ASTM Standards, ASTM International, West Conshohocken, PA, 2012	− Length and width are significantly greater than their thickness, including foils, sheets, and pads; − Unconsolidated particulate material that is not conveyed by scoops or similar equipment; − Closed, such as pillows and booms that confine the sorbent by using an external permeable fabric or mesh; − These include pompoms that have an open structure that allows the penetration of high-viscosity oils.	[7,8]
NF T90-362: Water and soil pollution control products—Classification of sorbents	Division based on selectivity: − Floating hydrophobic—designed to recover non-polar contaminants, used both on water surfaces and on land; − Hydrophilic—designed to recover polar and non-polar products for use only on hardened surfaces. Division based on form: − Loose (type A)—composed of particles without any connections between them; they come in the form of powders or short fibers of mineral origin (e.g., vermiculite), plant origin (e.g., sawdust, peat) or synthetic origin (e.g., PE, PS, PP); − Mats (type B)—mats or sheets—flexible products with a thickness of less than or equal to 3 mm, the length and width of which, although less than 1 m, are much greater than their thickness (a ratio of at least 10 between width/length and thickness), e.g., felts, polypropylene sheets; − Rolls (type C)—also known as blankets, these are products that can be up to several dozen meters long, are usually made of nonwoven fibers (felts), and can be reinforced with filament; − Pillows (type D)—flexible products in which the absorbent material is contained in a permeable envelope that is sufficiently resistant during use as is; the length is much greater than the thickness and is less than a meter; − Hoses (type E)—loose sorbent material is contained in a very oil-permeable envelope; the length is much greater than the other dimensions and exceeds 1 m; some models, designed for use on water, have a flexible ballast band that improves their ability to contain floating dirt; − Mops/pompons (type F)—these consist of threads of similar strands connected together, creating a light, open structure; − Special products (Type G)—products that do not fit into the above categories, A to F, e.g., a solid block of absorbent material to be placed under machinery to absorb drips.	[9,10]
Regulation of the Minister of Internal Affairs and Administration of 31 October 2022 on the list of products used to ensure public safety or protect health, life and property, as well as the principles of issuing approvals for use of these products (Journal of Laws 2022, item 2282)	Division according to sorption capacity: − Used on solid surfaces; − Used on water surfaces.	[11]

**Table 2:** Analysis of parameters for approved sorbents.

Parameter	Requirement
Oil absorption capacity	We required ≥50% by weight of the sorbent for diesel fuel meeting the requirements of EN 590. Measurement error of the method used: +/−3.74%
Sieve analysis	According to ISO 2591-1:2000, we used the dry sieving method, with the following range of sieves: 40 µm, 63 µm, 125 µm, 4 mm. The content of fractions with a grain size above 4 mm and fractions with a grain size smaller than 0.125 mm cannot differ by more than ±10% of the value declared by the manufacturer. Measurement error of the method used: +/−0.22%
Chemical inactivity	The chemical passivity test was performed during the oil absorption capacity test by observing four aspects, i.e., (i) gas release (organoleptic observation of smoke or change in smell), (ii) change in the color of the absorbed liquid, (iii) an increase in the temperature of the absorbed medium, (iv) change in the form of the sorbent. The sorbent cannot enter into chemical reactions with the absorbed substances. Organoleptic examination. Temperature measurement error: +/−0.2 °C
Buoyancy	This is assessed according to the method developed by the Laboratory of Fire Extinguishing Devices and Agents BU, which is within the scope of PCA accreditation (AB060) [16]—a sample previously tested for oil absorption capacity is used for testing. After the required time has elapsed, the amount of submerged sorbent should be assessed. This amount should not exceed 5% of the sorbent subjected to testing. In total, 95% of the sorbent used on water surfaces, both ready for use and used, fully saturated with a reference hydrocarbon according to EN 590, remains on the surface of standing water for 24 h. The requirement applies only to sorbents for collecting contaminants from water surfaces. Organoleptic examination
Bulk density	According to PN-C-04532:1980 method B, the bulk density value expressed in g/L cannot differ by more than ±10% of the manufacturer’s declared value. The requirement applies only to bulk sorbent. Measurement error of the method used: +/−0.95%

**Table 3:** Results of analyses of sorption materials obtained from waste in relation to meeting legal requirements in accordance with [13].

Sorption Material	Chemical Inertness	Buoyancy	Bulk Density [g/L]	Grain Composition
Polyurethane foam (PU)	Passive	Floating	52.66	<0.125 mm: 4.95% wt.; >4.0 mm: 0.05% wt.
Coffee grounds	Passive	Non-floating	341.49	<0.125 mm: 4.88% wt.; >4.0 mm: 0% wt.
Viscose–cellulose sorbent	Passive	Non-floating	NA *	NA *
Coconut fiber	Passive	Non-floating	NA *	NA *

* NA, not applicable—no work is being carried out in this area due to the fact that the requirement only applies to bulk sorbents.

**Table 4:** Comparison of the absorption capacity of selected commercial sorbents and those obtained from various wastes.

Sorptio Material	Sorption Capacity [% wt]	Notes	Ref.
*Commercial sorbents*			
Compakt	91.0	t = 10 min soaking and draining; Westinghouse method	[23]
ECOBARK	104.0		
Quartz sand	21.0		
*Waste sorbents*			
Viscose–cellulose sorbent	796.8 *–1191.2 **	t = 20 min soaking and draining; ambient temperature 20.5 ± 1 °C	Measured in this study
Polyurethane foam	601.9 *–981.1 **		
Coconut fibers	110.5 *–144.1 **		
Coffee grounds	129.7 *–140.9 **		
Corn with cotton	3790	t = 15 min; test: 0.5 L of artificial seawater with a concentration of 3.5% NaCl; layer of diesel oil/crude oil with different thicknesses of 1, 3, and 5 mL; ambient temperature 25 ± 1 °C	[24]
Barley straw	780–1220	t = 15 min soaking, 5 min draining; particle size 250 μm; ambient temperature 25 ± 1 °C; test: oil + seawater, film thickness 5 mm	[25]
Banana peels	531–663	t = 15 min soaking and draining; particle size 0.3625 mm; ambient temperature 25 °C	[3]
Onion peels	45.5	t = 90, 30, and 30 s contact; test: 0.6 g crude oil in physiological saline solution; 750 mL 0.5 M NaCl; ambient temperature 30 °C	[26]
Garlic peels	38.5		

* data for diesel oil; ** data for machine oil.

**Table 5:** Proposed purpose of the analyzed sorption materials for the elimination of contamination.

Product	Purpose
Polyurethane foam (PU)	A product designed to eliminate oil and petroleum spills in industrial halls, garages, and underground car parks and in open areas
Coffee grounds	A product designed to eliminate oil and petroleum spills in industrial halls, garages, and underground car parks and in open areas
Viscose–cellulose sorbent	A product designed to collect water, oil, and petroleum contaminants from solid surfaces
Coconut fiber	Designed for collecting oil and petroleum contamination from solid surfaces. The product is used in the absorption of oils, gasoline, kerosene, cutting fluids, cooling fluids, emulsions, rinsing fluids and paints

## Data Availability

The original contributions presented in this study are included in the article. Further inquiries can be directed to the corresponding author.
